# AuthorSynth: a collaboration network and behaviorally-based visualization tool of activation reports from the neuroscience literature

**DOI:** 10.3389/fninf.2015.00006

**Published:** 2015-03-25

**Authors:** Vanessa V. Sochat

**Affiliations:** Graduate Program in Biomedical Informatics, Stanford UniversityStanford, CA, USA

**Keywords:** functional magnetic resonance imaging (fMRI), collaboration networks, meta-analysis, web applications, machine learning

## Abstract

Targeted collaboration is becoming more challenging with the ever-increasing number of publications, conferences, and academic responsibilities that the modern-day researcher must synthesize. Specifically, the field of neuroimaging had roughly 10,000 new papers in PubMed for the year 2013, presenting tens of thousands of international authors, each a potential collaborator working on some sub-domain in the field. To remove the burden of synthesizing an entire corpus of publications, talks, and conference interactions to find and assess collaborations, we combine meta-analytical neuroimaging informatics methods with machine learning and network analysis toward this goal. We present “AuthorSynth,”^1^ a novel application prototype that includes (1) a collaboration network to identify researchers with similar results reported in the literature; and (2) a 2D plot—”brain lattice”—to visually summarize a single author’s contribution to the field, and allow for searching of authors based on behavioral terms. This method capitalizes on intelligent synthesis of the neuroimaging literature, and demonstrates that data-driven approaches can be used to confirm existing collaborations, reveal potential ones, and identify gaps in published knowledge. We believe this tool exemplifies how methods from neuroimaging informatics can better inform researchers about progress and knowledge in the field, and enhance the modern workflow of finding collaborations.

## Introduction

Neuroimaging has provided huge insight to the underpinnings of the human brain, with roughly 10,000 papers in PubMed in 2013, each associated with multiple authors spanning across the globe (ISI Web of Knowledge, 2010). Essential to the generation of this knowledge is collaboration, which allows for the efficient and intelligent synthesis of resources, time, and expertise. However for imaging scientists-already challenged with simultaneously conducting research, writing and reviewing papers, and securing funding-staying on top of the social and networking aspect of the field may not take priority. While conferences and social media offer a portal to find collaborators, it takes substantial effort for any single researcher to evaluate the work of those he or she encounters, and many working on similar things are more unlikely than likely to cross paths. While redundancy in research is essential for validation of biological findings, having a clear understanding of whom is working on similar problems and of gaps in knowledge is essential to moving the field forward. The responsibility of the researcher to keep up with publications, conferences, and potential colleagues is turning into an overwhelming burden, one that could be ameliorated by better methods to synthesize the growing collaboration network.

A more comprehensive strategy for pursuing efficient collaboration would harness the results themselves: peer-reviewed publications are a good reflection of an author’s current and historical body of work. Thus, our goal in this work was two-fold. First, we aimed to harness the bibliome of neuroimaging literature to summarize the work of the top researchers in the field, where each researcher has defined a brain map that shows regional contribution. We concurrently developed a novel visualization strategy to show these results. Second, we used network analysis to generate a network of actual collaborations onto which we could map these results. We have developed a web interface, “AuthorSynth,” for exploration of author contribution and collaborations. Our method is a novel meta-analytical approach that offers neuroimaging researchers a data-driven, intuitive summarization of published work, and an avenue to find new opportunities.

## Methods

**Figure 1:**
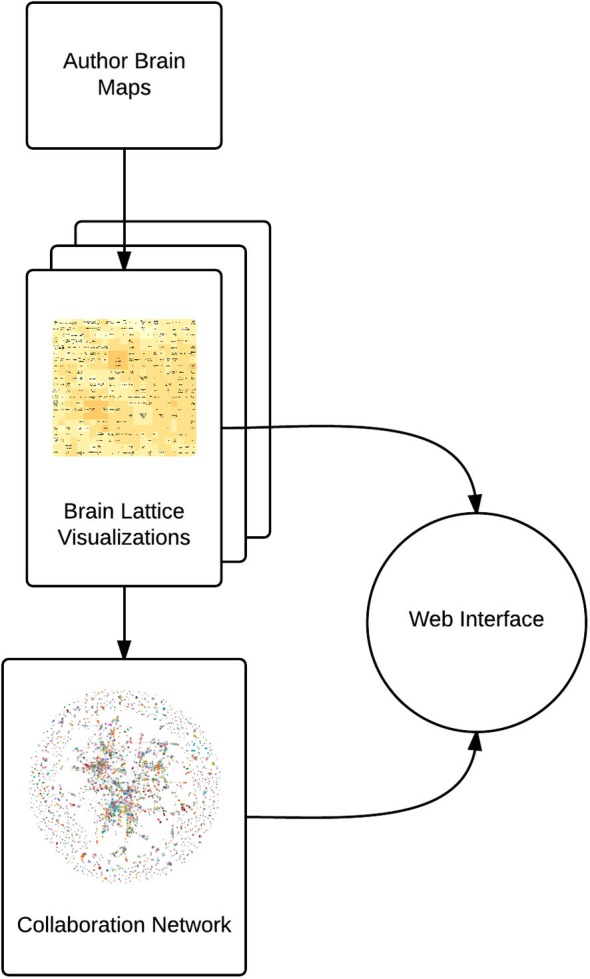
**Summary of methods**. We use NeuroSynth to generate a brain map for each author (Author Brain Maps), summarize each author’s contributions (Brain Lattice Visualizations), and then map this data onto a collaboration network (Collaboration Network). We combine these components into an interactive web interface.^4^

AuthorSynth is an interactive web tool to visualize existing and potential collaborations between neuroscience authors. Generation of this tool involved mining of the neuroscience bibliome to generate a brain map for each author (Section Creating Brain Maps to Spatially Summarize Top Contributions in Human Brain), summarizing these maps in a novel two dimensional grid—a “brain lattice”—that links regional activation to behaviorally relevant terms (Section Mapping Author Brain Maps to Psychologically-Relevant Brain Maps), identifying authors with similar published work based on an assessment of these maps (Section Identifying Similar Authors in the Brain Lattice), generating a network of actual collaborations onto which to map this data (Section Generation of Collaboration Network), and finally, combining these components into an interactive web interface (Section The AuthorSynth Web Portal). A summary of our methods is included in Figure 1, and all code to implement our methods^2^ and the interface itself^3^ has been made publicly available.

### Creating Brain Maps to Spatially Summarize Top Research Contributions in Human Brain

The NeuroSynth database is a comprehensive collection of activation points from the top neuroimaging journals, covering 5,809 articles across 17 journals at the time of our study (Yarkoni et al., 2011). NeuroSynth organizes reports of significant brain activation at the voxel level, and allows for the exploration of associations between these reports and behavioral terms reported in the text to generate brain maps that represent what the compendium of neuroscience literature has to say about a cognitive or psychologically-relevant term. The NeuroSynth algorithm works by mining the bibliome of neuroimaging studies to find the coordinates of voxels reported, “published activation coordinates,” in structured tables, and generating a 2 × 2 contingency table of counts for each voxel to indicate if activation is present or absent when a specific behavioral term is present or absent. A Chi-Square test of independence is used to determine if there is a significant dependence between the term and activation, and voxels that do not pass significance threshold (FDR-adjusted to account for multiple hypothesis testing at a threshold of 0.05) are zeroed out (Yarkoni et al., 2011). We aimed to use this resource not to link activation reports to behavioral terms, but rather to the authors that published them.

The authors represented in the NeuroSynth database represent a robust sample of neuroscience researchers, each associated with a set of neuroscience publications with activation reports, as detailed above. We extracted this list of 19,677 unique authors from the NeuroSynth database, as well as a complete list of PubMed identifiers associated with each author. We first reduced this list of authors to those who are likely to be principal investigators (PIs). We defined a PI as a neuroscience author appearing as last author for at least two papers (3,383), and we used this subset for our analysis. We used the NeuroSynth algorithm described above to generate corrected (FDR 0.05) brain maps with non-zero voxel values having an FDR corrected *p*-value that represents a significant dependence between the activation point and researcher. In the terminology widely used within the neuroimaging community, these images represent “reverse inference” maps in that they provide an index of the degree to which activation at every voxel is preferentially associated with the presence of a particular author relative to all other authors in the database. Following procedures in Yarkoni et al. (2011), the maps were spatially normalized to the Montreal Neurological Institute (MNI) Template, a standard space measured in cubic millimeters that allows for comparison of brain imaging data with different voxel dimensions. Brain maps were converted to *Z*-scores so that the values at each voxel represent a normalized version of the test described above. This procedure produced a brain map for each PI that represents his or her contribution to the literature based on compiled activation reports.

### Mapping Author Brain Maps to Psychologically-Relevant Brain Maps

To intelligently visualize the 3,383 brain maps generated in Section Creating Brain Maps to Spatially Summarize Top Research Contributions in Human Brain we developed a novel visualization strategy—the “brain lattice”—to map regional patterns of activation to a 2D space defined by behavioral terms (e.g., “anxiety” or “motor”). While overlaying the author brain maps on a standard brain would provide some degree of understanding about the regions studied to an expert in the field, a non-expert cannot easily obtain this understanding. We aimed for a simpler, more intuitive 2D visualization that would show the research being done by a PI as “hot” colors on a grid with behavioral terms labels. For example, an author brain map that has non-zero voxels in a part of motor cortex should produce hot colors on the brain lattice over regions related to motor terms, and a viewer does not need to understand the anatomical specificity of motor cortex to see this. The generation of this brain lattice required two steps. First, we used the NeuroSynth software package^5^ to generate FDR-corrected “reverse inference” activation maps for 525 psychologically relevant behavioral terms, using default parameter settings. Equivalently to the author brain maps, each non-zero voxel in our behavioral term maps indicates the voxel having a significant association with the behavioral term.

Second, we used a method from machine learning, the self-organizing map (SOM) as implemented with the kohonen package in R (Wehrens and Buydens, 2007) to generate the brain lattice. The SOM is an unsupervised method from machine learning that represents similarity in high-dimensional data by way of distance on a two dimensional grid. The SOM itself is a grid of nodes, each of which is associated with a vector of weights of equal length to the training data, which in our case were the 525 3D behavioral brain maps flattened into vectors. The weights of each node are initialized to random values in the same space as the training data, and subsequent training consists of choosing a vector at random from the training data, and matching it to the most similar node—called the “Best Matching Unit” (BMU)—as determined by Euclidean distance. We then define the BMU’s local neighborhood by way of an exponential decay function:

(1) $$\sigma (t)=\sigma_{0}\exp\left(-\frac{t}{\text{λ}}\right)\;t=1,\;2,\;3\ldots$$

where *σ*_0_ is the width of the lattice at time *t*_0_, *λ* is a time constant, and *t* is the iteration number.

This decay function decreases the width of the neighborhood as a function of time, until training is complete when the neighborhood is the same size as the BMU. During each iteration, the weights of the nodes that fall within this width of the BMU are adjusted according to the following equation:

(2) W(t+1)=W(t)+L(t) ​(V(t)−W(t))

where *t* again represents the iteration number of time set, and *L* is the learning rate. This equation can be interpreted to say that the weight of a node at the following time point is equal to the old weight plus a small percentage of the difference between the old weight and the training vector. This procedure ensures that nodes defined within the neighborhood are changed to be more similar to the matched training data. This entire procedure results in a brain lattice that has nodes representing “meta” brain maps of the behaviorally-based brain images directly assigned, and each node having some influence from brain maps assigned in the periphery. By labeling the nodes with the terms that describe the brain images, we achieve an understanding of how the term maps relate to one another, as similar brain images will be assigned with closer proximity or to the same node in the map. More powerfully, the weight vector corresponding to each node in this space that has been influenced by both its matched training vectors and local neighborhood can be reshaped back into a 3D image, allowing for an assessment of similarity of a new image to each node, and then a coloring of the map based on this similarity value.

The final step is to map the author brain maps (Section Creating Brain Maps to Spatially Summarize Top Research Contributions in Human Brain) onto this 2D space. As was stated above, each node has associated with it a vector of weights that can be reshaped back into a 3D image to represent the node. We can then, for each author brain map, calculate the similarity of the author map to each node using the cosine similarity. The choice for the size of the map is arbitrary, and we chose the maximum dimension (22 × 23) that would ensure no resampling of images for training for a total of 506 nodes. We generated a matrix, C of size 3,383 × 506, with each value *C*[*a*,*b*] for a given author *a*, and a node, *b*, corresponding to the cosine similarity between the author brain map vector, *A*, and each of the 506 node map vectors, *B*. The cosine similarity, cos(θ), is defined as:

(3) $$\cos(\theta )=\sum_{i=1}^{n}\frac{A_{i}\times B_{i}}{\sqrt{\sum_{i=1}^{n}A_{i}^{2}}\times \sqrt{\sum_{i=1}^{n}B_{i}^{2}}}$$

This metric measures the angle between the two vectors, meaning that for each author brain map and a brain lattice of size 22 × 23 (*N* = 506 nodes), we produce 506 match scores corresponding to nodes in the brain lattice. We can then map these scores to a color gradient to produce a final brain lattice image for each author. It follows that authors with similar match scores to the nodes in the map result in similar brain lattices, and that these similar brain lattices are reflective of having similar published activation coordinates.

### Identifying Similar Authors in the Brain Lattice

We identified groups of similar researchers by making a pairwise assessment of the row vectors of author match scores to the SOM in matrix C described above. We defined a distance matrix, *M*, of size 3,383 by 3,383 where each value *M*⌊*a_i_*,*a_j_*⌋ is the Euclidean distance between author SOM match scores in vectors *C*[*a_i_*,] and *C*[*a_j_*,]. The goal of this matrix was two-fold. First, we could look at the 3,383 similarity scores for a given author and sort these scores in a descending fashion to generate a ranked list of similar authors for the single author. Second, we could use hierarchical clustering to generate a dendrogram that shows groups of similar authors. In both cases, as the similarity is based on having similar brain lattices, this is indicative of having similar activation reports. We used the hclust function in R (Müllner, 2013) with standard Euclidean distance of matrix *M* to produce this clustering. As it is not clear where to cut the tree to define final groups, we generated groups based on cutting the tree at a selected range of heights (1, 2, 2.5, 3, 3.5, 4) as well as number of groups (5, 10, 15, 20, 25, 30, 50, 60, 70, 80, 90, 100) to allow for an interactive thresholding of the tree and definition of groups. These groupings can then be mapped onto a network of actual collaborations, as discussed in the next section.

### Generation of Collaboration Network

Comparison of the brain lattices generated in the previous section allows us to identify researchers who are conducting similar work based on activation reports in the literature; however, it does not tell us which researchers are actually working together. Our second goal was to generate a network of actual collaborations onto which to map this knowledge. To generate a network of actual collaborations, we first created a list of all pairs of neuroscientists in the NeuroSynth database that appear together on at least one paper, and annotated this list with the number of papers the authors published together. We then generated a force directed graph using d3 (Bostock et al., 2011), where a node is defined for each of the 3,383 PI and an edge is introduced whenever two PIs have two or more publications together. We used a JavaScript library, “Data Driven Documents” (d3) to generate this network (Bostock et al., 2011). This network can then be interactively annotated with a color mapping that reflects the groupings generated in Section Identifying Similar Authors in the Brain Lattice.

### The AuthorSynth Web Portal

We have compiled the author brain lattices and collaboration network into an interactive web interface, AuthorSynth, available at http://www.vbmis.com/bmi/authorSynth using standard hyper transfer markup language (HTML), cascading style sheets (CSS), and JavaScript (W3 Consortium, 1998a,b; Flanagan, 2002). A user of the interface can explore the collaboration network as a force directed graph, search for researchers within the map, and interactively threshold the map. As previously stated, the network defines collaborations as links between 3,383 PIs with two or more publications together. The coloring of the map reflects groupings determined by similarity of brain lattices, or regional activation reports (Section Identifying Similar Authors in the Brain Lattice). A small cluster of connected nodes that are the same color indicates that authors with a similar body of published work are actually working together, while a color spread across many regions of the map indicates that authors with similar published work are not actually working together. A user of the interface can also search for a particular author to view a “Single Author Page” that includes a single author brain lattice, a ranked list of researchers with similar brain lattices, and publications alongside activation points used in the analysis. At each spot in the ranked list we define a “collaboration score” that reflects the percentage of researchers up to that point that the author has actually worked with, and a “match score” (described below) to indicate the similarity of the potential collaborator brain map to the author brain map. A circle next to either author in the ranked list is sized to reflect the “collaboration score,” and positioned along the *x*-axis to define the “match score”. Using these circles, we highlight actual collaborators in red, and clicking the circles links to the “Single Author Page” for the author in question. Finally, we provide a second entry point, a single interactive brain lattice, from which the user can click on a node to return a list of authors most highly matched to the node. For example, a user interested in finding neuroscience researchers that have done work related to auditory processing could click on the node labeled with “auditory” to return a ranked list of authors most highly matched to that node. We define “most highly matched” as authors having a match score in the top 2.5% of all match scores (*Z* score > 1.96), and limit the result to return 50 matches.

## Results

### Creating Brain Maps to Spatially Summarize Top Research Contributions in Human Brain

**Figure 2:**
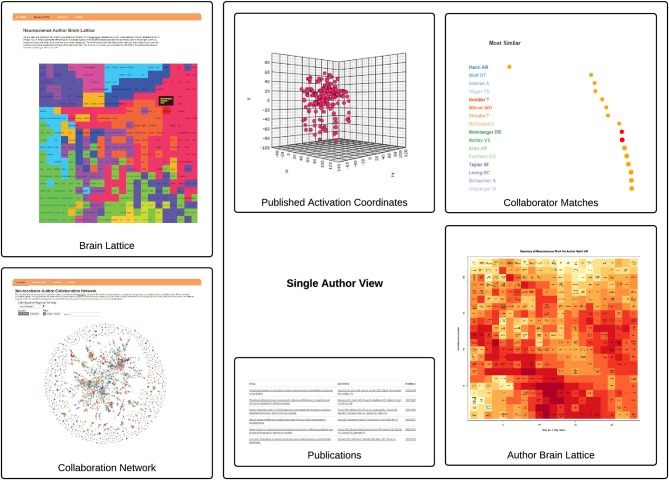
**The AuthorSynth Web Interface**. The user can enter AuthorSynth to explore the data through a behaviorally-based brain lattice (“Brain Lattice,” top left), or through a collaboration network (“Collaboration Network,” bottom left). Investigation of a single author (“Single Author View”) from the network or as a high match in the brain lattice provides a single author brain lattice (“Author Brain Lattice”), a ranked list of authors with similar brain maps (“Collaborator Matches”), activation coordinates provided by the NeuroSynth database used to derive the brain maps (“Activation Coordinates”), alongside the papers from which they were derived (“Publications”). Red circles in the list indicate actual collaborations, and “hot” spots in the brain lattice indicate published work similar to those behavioral terms in the map. This visualization is intended to provide a high level view—for a detailed review please visit.^6^

We generated a brain map for each of the 3,838 PIs in the NeuroSynth database. The activation coordinates that were used to generate a single author brain map are shown alongside a subset of papers from which they are derived in Figure 2.

### Mapping Author Brainmaps to Psychologically-Relevant Brainmaps

**Figure 3:**
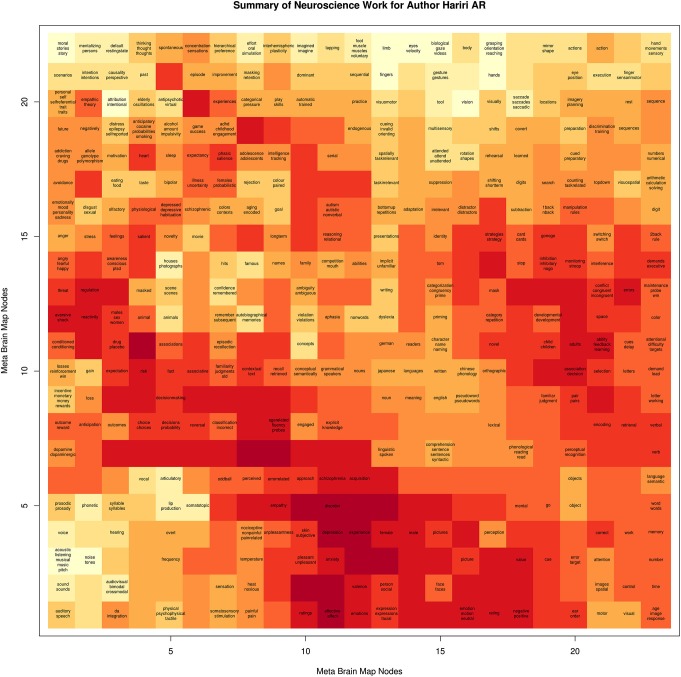
**Example of Brain Lattice**. An example “brain lattice” for researcher Ahmad R Hariri, Director of the Laboratory of NeuroGenetics at Duke University. Professor Hariri studies neural circuits supporting threat and reward processing, and so his map reflects this work with “hot” spots around terms related to depression, anxiety, emotional reactivity, and decision making.

We generated behavioral brain term maps for a set of 525 psychologically-relevant terms included in the base NeuroSynth data repository (version 0.x) (Yarkoni et al., 2011). We used the SOM to map these 3D images onto a 2D space, as described in methods Section Mapping Author Brain Maps to Psychologically-Relevant Brain Maps. The finished brain lattice, colored to show similar portions of the map, is shown in the top left panel of Figure 2. Finally, we mapped each author brain map to this space, and projected match scores onto a color gradient to define a unique mapping for each author, with “hot” colors corresponding to higher match scores, and cooler colors to lower match scores. A detailed brain lattice defined for researcher Ahmad Hariri is included in Figure 3.

### Identifying Similar Authors in the Brain Lattice

We generated pairwise similarity scores between author brain lattice match scores to generate a ranked list of similar authors for each single author. An example list is shown in the “Single Author View” in Figure 2. We clustered these same brain lattice match scores to identify groups of similar authors, varying the threshold to define a wide range of groupings (Section Identifying Similar Authors in the Brain Lattice). These groupings were then used to color the collaboration network.

### Generation of Collaboration Network

The resulting network has 3,383 nodes, and 3,129 links. The purpose of this network is to visually show authors that are actually working together, indicated by having a link between them, vs. those with similar published work, indicated by having an equivalent color. This interactive network is available as an online resource,^7^ and also shown as a static image in Figure 2.

### The AuthorSynth Web Portal

A summary of the AuthorSynth interface is described in Figure 2. The web portal presents the user with two methods of entry to explore the data: a dynamic collaboration network, and an interactive brain lattice. From the interactive brain lattice, the user can click a behavioral term to return a list of authors that are most highly matched to the node, meaning that the brain map that represents the author’s published work is similar to the NeuroSynth brain map for that term. From the collaboration network the user can search for and go directly to a “Single Author View” page, which includes an “Author Brain Lattice” (Section The Single Author Brain Lattice), a list of similar authors (Section Similar Authors Based on Published Activation Coordinates), and publications alongside activation coordinates that were used in the analysis (Section Publications and Activation Coordinates). The collaboration network has links between PI nodes that represent actual collaborations, and coloring that reflects similarity of published activation reports.

#### The Single Author Brain Lattice

The single author brain lattice is equivalent in structure to the portal brain lattice, however instead of being colored to indicate similar portions of the map, it is colored by matching scores of the author brain map to each node. An area of the map that is “hot” is indicative that the author’s published work is highly similar to the node in the SOM, and inspection of the behavioral-terms around this node provides a behavioral interpretation of the author’s work. This single author brain lattice can also be interactively clicked to return authors highly matched to the node of interest.

#### Similar Authors Based on Published Activation Coordinates

The ranked list of similar authors defined based on having similar brain lattices and therefore similar published work (Section Identifying Similar Authors in the Brain Lattice) is provided as a tab on the “Single Author View” page. The goal of this ranked list is to identify similar authors based on published work, and the underlying assumption is that PIs who study similar brain regions could potentially work together, or minimally know about one another. The author in question is at the top of the list, followed by a list of other authors in the database ordered by most to least similar. Next to each author name is a circle plotted along an *x*-axis that represents the similarity score: circles farther to the right correspond to lower similarity scores. The circles are colored red (indicative of a collaboration), and orange (indicative of no collaboration) so that the user of the interface can quickly assess if the author in question is collaborating with researchers with similar published work. A user of the interface can click on any of these circles to view the “Single Author View” for the author in question.

#### Publications and Activation Coordinates

The “Single Author View” includes a tab for the author’s publications, including titles, full lists of authors, and links to the articles themselves. The page also includes a 3D plot of the activation coordinates described in those papers that were used in the analysis.

## Discussion

The AuthorSynth collaboration network provides a straight-forward visualization of researchers with similar work, and the brain lattices associated with each author describe what that work encompasses. This initial interface has several use cases. A PI can find his or her page in the database, and quickly make an assessment of what portion of the individuals doing similar work he or she has actually published with. The PI can then explore the work of these similar authors, and potentially find new collaborators. A scholar interested in finding PIs that do research of a specific type can enter through the brain lattice portal, and return a list of PIs with work that matches a behavioral term of interest.

The goal of this work was to introduce the AuthorSynth scaffolding that can be built upon for more advanced analyses. While a complete evaluation of the network itself is outside of the scope of this technology report, there are several observations that deserve mention, along with future plans for more advanced network analysis.

### Future Improvements and Applications

It is interesting that the coloring of the collaboration network, indicative of similarity of author brain maps and thus published work, does not more cleanly map onto the collaboration network. We hypothesize that this may be an indication that many authors conducting similar research are in fact not working together, and further, that introducing additional meta-information about authors could reveal patterns to explain the groupings. Our method does not take author order or institution into account when defining collaboration between PIs, and it would be interesting to think about how to incorporate this into the algorithm as some sort of weight. A collaboration of a PI with an author as first author (indicative of being the primary author behind the work) could arguably be more highly weighted than a collaboration of a PI with an author as middle author, for example. Likewise, collaboration between authors at the same institution may not be as interesting as collaboration between institutions, and it would be interesting to extract additional author meta-information to color the collaboration network.

The future applications beyond collaboration discovery to apply the AuthorSynth scaffolding are immense. We are interested in investigating “gaps” in the study of brain based on published coordinates. With the growing availability of whole-brain statistical maps (Gorgolewski et al., 2014), it might eventually be possible to determine if this is an artifact of the coordinate-based approach, and if not, point researchers in the direction of areas that warrant further study. It will be interesting to identify if there are strong associations between published coordinates and institution, or if there is some quality (meta-information) of a researcher that is more strongly associated with a pattern of published coordinates. It would be fairly feasible to generate institution-specific sub-networks, and provide feedback to institutions about inter-department collaboration. By mapping these collaborations and funding sources onto this publication network, we can better quantify the productivity of the neuroscience community as a whole, and provide meaningful feedback for future allocation of funds.

### Limitations

This tool has several limitations. First, we were limited by the number of studies present in the NeuroSynth database at the time of our analysis. NeuroSynth includes the top journals that report exclusively neuroimaging results (Yarkoni et al., 2011), as this set is limited to publications that report activation values in structured HTML tables that can be mined by an algorithm. As more journals adopt a web-interface with articles in HTML format, and the NeuroSynth text mining algorithm is modified to include these new formats, the database will allow for a more substantial compendium of publications that can generate a more robust network.

#### Network Node Definition

We limited our network to PIs with at least two publications, as a network with all 19,677 authors in the NeuroSynth database was computationally infeasible to render in a browser. This introduces an assumption into the design of the tool that its users will be most interested in these individuals, and we would like to develop a visualization strategy that would allow for exploration of the larger network.

#### Author Name Disambiguation

A final limitation is the possibility of disambiguation of PI author names in this set that would introduce a small amount of noise into our network. While we recognize this larger issue (Smalheiser and Torvik, 2009), the focus of this initial work is on the tool infrastructure, and we are pursuing further work focused on analysis of the network itself.

## Conclusion

We have created AuthorSynth, a novel web interface prototype that both summarizes authors’ work based on activation reports, and qualitatively visualizes the work in an intuitive way—on a 2D, colorful brain lattice. This work demonstrates that the integration of meta-analytical methods with machine learning and network analysis can provide what we believe to be a useful, relevant way to identify researchers with similar published work. While the focus of this initial work is on the development of the tool’s infrastructure, we are excited about future work to conduct more advanced network analysis to drive more advanced applications. The prototype web interface should be of interest to researchers with a substantial enough body of work to have publications in the database, and to researchers that are searching for collaboration opportunities. We believe this work is an important step in the right direction to better harness methods from neuroimaging informatics to assist with collaboration to move the field move forward, and we are excited to pursue further analysis with our infrastructure.

## Data Availability

Complete code for methods in Python and R^8^, and the interface itself^9^ in HTML, CSS, and JavaScript has been made publicly available.

## Conflict of Interest Statement

The author declares that the research was conducted in the absence of any commercial or financial relationships that could be construed as a potential conflict of interest.

## Footnotes

^2^https://github.com/vsoch/authorSynth

^3^http://www.vbmis.com/bmi/authorSynth

^4^http://www.vbmis.com/bmi/authorSynth

^5^http://github.com/neurosynth/neurosynth

^6^http://www.vbmis.com/bmi/authorSynth

^7^http://www.vbmis.com/bmi/authorSynth/network.html

^8^https://github.com/vsoch/authorSynth

^9^http://www.vbmis.com/bmi/authorSynth
